# Supplementary material for: The Systems Biology Research Tool: evolvable open-source software
Source: BMC Syst Biol. 2008 Jun 29;2:55. doi: 10.1186/1752-0509-2-55 (PMC2446383; doi:10.1186/1752-0509-2-55)
Supplement: Additional file 1 — SBRT Archive. An archive of the current version of the Systems Biology Research Tool. [file 1752-0509-2-55-S1.zip › sbrt-1.4.0/doc/developers_guide/api/sbrt/kernel/math/class-use/LinearComb.html]

Uses of Interface sbrt.kernel.math.LinearComb


|  |  |  |
| --- | --- | --- |
| **Overview** \| **Package** \| **Class** \| **Use** \| **Tree** \| **Deprecated** \| **Index** \| **Help** | |  |
| PREV   NEXT | **FRAMES**    **NO FRAMES**     **All Classes** |  |


---


## **Uses of Interface sbrt.kernel.math.LinearComb**

| Packages that use LinearComb | |
| --- | --- |
| **sbrt.ext.cplex** | Provides a class that allows communication with CPLEX. |
| **sbrt.ext.glpk** | Provides a class that allows communication with GLPK. |
| **sbrt.ext.mma.algebra** | Provides classes that allow communication with Mathematica for the purposes of algebra. |
| **sbrt.kernel.fba** | Provides classes and interfaces for creating objects relevant to Flux Balance Analysis. |
| **sbrt.kernel.fba.comp** | Provides classes and interfaces for performing Flux Balance Analysis computations. |
| **sbrt.kernel.fba.proc** | Provides classes and interfaces for executing Flux Balance Analysis processes. |
| **sbrt.kernel.math** | Provides classes and interfaces for creating objects relevant to mathematics. |
| **sbrt.kernel.math.algebra** | Provides classes and interfaces for creating objects relevant to algebra. |
| **sbrt.kernel.math.algebra.proc** | Provides classes and interfaces for executing algebra-related processes. |
| **sbrt.kernel.math.geom** | Provides classes and interfaces for creating objects relevant to computational geometry. |
| **sbrt.kernel.math.optimization** | Provides classes and interfaces for creating objects relevant to solving optimization problems. |
| **sbrt.shell.io.fba** | Provides classes and interfaces for reading and writing files relevant to Flux Balance Analysis. |
| **sbrt.shell.mng.algebra** | Provides classes for creating algebra process managers. |
| **sbrt.shell.mng.fba** | Provides classes and interfaces for creating Flux Balance Analysis process managers. |
| **sbrt.shell.mng.utilities** | Provides classes and interfaces for creating mathematical process managers. |
| **sbrt.shell.text.fba** | Provides classes and interfaces for defining formats for objects relevant to Flux Balance Analysis. |
| **sbrt.shell.text.math** | Provides classes and interfaces for defining formats for mathematical objects. |

Uses of LinearComb in sbrt.ext.cplex

| Methods in sbrt.ext.cplex with parameters of type LinearComb | |
| --- | --- |
| `void` | `CplexSolver.setConstraint(LinearComb<? extends C> linComb, Interval c)`             Sets a constraint on the provided mathematical expression of column variables. |
| `void` | `CplexSolver.setObjectiveFunction(LinearComb<? extends C> objective)`             Sets the objective function to the provided mathematical expression of column variables. |
| `void` | `CplexSolver.setObjectiveFunction(LinearComb<? extends C> objective, OptimizationSense sense)`             Sets the objective function and optimization sense to the provided values. |

Uses of LinearComb in sbrt.ext.glpk

| Methods in sbrt.ext.glpk with parameters of type LinearComb | |
| --- | --- |
| `void` | `GlpkSolver.setConstraint(LinearComb<? extends C> expression, Interval c)`             Sets a constraint on the provided mathematical expression of column variables. |
| `void` | `GlpkSolver.setObjectiveFunction(LinearComb<? extends C> expression)`             Sets the objective function to the provided mathematical expression of column variables. |
| `void` | `GlpkSolver.setObjectiveFunction(LinearComb<? extends C> expression, OptimizationSense sense)`             Sets the objective function and optimization sense to the provided values. |

Uses of LinearComb in sbrt.ext.mma.algebra

| Methods in sbrt.ext.mma.algebra that return types with arguments of type LinearComb | |
| --- | --- |
| `protected static java.util.Map<java.lang.String,LinearComb<java.lang.String>>` | `MMaExprInterpretter.interpretSolution(com.wolfram.jlink.Expr solution)`             Converts the provided Mathematica expression to a `Map`. |

| Method parameters in sbrt.ext.mma.algebra with type arguments of type LinearComb | |
| --- | --- |
| `LinearSystemSolution<V>` | `MathematicaSolver.solve(java.util.Map<LinearComb<V>,java.lang.Double> equations)`             Computes and returns a solution to the provided system of linear equations. |

Uses of LinearComb in sbrt.kernel.fba

| Methods in sbrt.kernel.fba that return LinearComb | |
| --- | --- |
| `LinearComb<java.lang.String>` | `ExchangeRxn.getProducts()`             Returns an empty linear combination. |
| `LinearComb<java.lang.String>` | `AbstractRxn.getProducts()`             Returns the products of this reaction. |
| `LinearComb<java.lang.String>` | `SourceRxn.getProducts()`             Returns a single-term linear combination. |
| `LinearComb<java.lang.String>` | `ChemRxn.getProducts()`             Returns the products of this chemical reaction as a linear combination of chemical species. |
| `LinearComb<java.lang.String>` | `ExchangeRxn.getReactants()`             Returns an empty linear combination. |
| `LinearComb<java.lang.String>` | `AbstractRxn.getReactants()`             Returns the reactants of this reaction. |
| `LinearComb<java.lang.String>` | `SinkRxn.getReactants()`             Returns a single-term linear combination. |
| `LinearComb<java.lang.String>` | `ChemRxn.getReactants()`             Returns the reactants of this chemical reaction as a linear combination of chemical species. |

| Methods in sbrt.kernel.fba with parameters of type LinearComb | |
| --- | --- |
| `protected  double` | `AbstractRxn.getRatio(LinearComb<?> expr1, LinearComb<?> expr2)`             Returns the ratio of the coefficients in the provided expressions. |
| `protected  double` | `AbstractRxn.getRatio(LinearComb<?> expr1, LinearComb<?> expr2)`             Returns the ratio of the coefficients in the provided expressions. |

Constructors in sbrt.kernel.fba with parameters of type LinearComb

`AbstractRxn(LinearComb<java.lang.String> reactants,
LinearComb<java.lang.String> products)`
  
          Constructs a new chemical reaction from the provided reactants
and products with the given name.

`AbstractRxn(LinearComb<java.lang.String> reactants,
LinearComb<java.lang.String> products)`
  
          Constructs a new chemical reaction from the provided reactants
and products with the given name.

`IrrevRxn(LinearComb<java.lang.String> reactants,
LinearComb<java.lang.String> products)`
  
          Constructs a new irreversible reaction from the provided reactants and
products.

`IrrevRxn(LinearComb<java.lang.String> reactants,
LinearComb<java.lang.String> products)`
  
          Constructs a new irreversible reaction from the provided reactants and
products.

`ReversibleRxn(LinearComb<java.lang.String> reactants,
LinearComb<java.lang.String> products)`
  
          Constructs a new reversible reaction from the provided reactants and
products.

`ReversibleRxn(LinearComb<java.lang.String> reactants,
LinearComb<java.lang.String> products)`
  
          Constructs a new reversible reaction from the provided reactants and
products.

Uses of LinearComb in sbrt.kernel.fba.comp

| Methods in sbrt.kernel.fba.comp that return LinearComb | |
| --- | --- |
| `protected <V> LinearComb<V>` | `FbaOptComputer.getExpr(java.util.Collection<V> columnVariables)`             Converts a collection of column variables to a linear combination. |
| `protected <V> LinearComb<V>` | `FbaOptComputer.getExpr(V columnVariable)`             Converts a column variable to a single-term linear combination. |
| `protected  LinearComb<java.lang.String>` | `AbstractCvComputer.getRxnExpr(java.util.Collection<java.lang.String> reactionNames)`             Converts a collection of reaction names to a linear combination. |
| `protected  LinearComb<java.lang.String>` | `FbaOptComputer.getRxnExpr(java.util.Collection<java.lang.String> rxnNames)`             Converts a collection of reaction names to a linear combination. |
| `protected  LinearComb<java.lang.String>` | `AbstractCvComputer.getRxnExpr(java.lang.String reactionName)`             Converts a reaction name to a single-term linear combination. |
| `protected  LinearComb<java.lang.String>` | `FbaOptComputer.getRxnExpr(java.lang.String reactionName)`             Converts a reaction name to a single-term linear combination. |

| Methods in sbrt.kernel.fba.comp that return types with arguments of type LinearComb | |
| --- | --- |
| `java.util.Map<java.lang.String,java.util.Set<LinearComb<java.lang.String>>>` | `FluxCapComputer.getFluxCaps()`             Computes and returns all flux caps. |
| `java.util.Map<LinearComb<java.lang.String>,Interval>` | `CdHarFluxVectorComputer.getRxnExprConstraints()`             Returns all current flux expression constraints. |

| Methods in sbrt.kernel.fba.comp with parameters of type LinearComb | |
| --- | --- |
| `void` | `CdHarFluxVectorComputer.restoreRxnExprConstraint(LinearComb<java.lang.String> expr)`             Restores the constraint on the provided flux expression to the value contained in the fluxome. |
| `void` | `CdHarFluxVectorComputer.setRxnExprConstraint(LinearComb<java.lang.String> rxnExpr, Interval c)`             Changes the constraint on the provided linear combination of fluxes. |

Constructor parameters in sbrt.kernel.fba.comp with type arguments of type LinearComb

`FastMsFluxRangeComputer(FbaOptComputer computer,
java.util.Map<java.lang.String,java.util.Set<LinearComb<java.lang.String>>> fluxCaps,
LinearSystemSolution<java.lang.String> systemSolution,
double zeroCutoff)`
  
          Constructs a new flux interval computer.

`FluxCapComputer(Fluxome fluxome,
java.util.Collection<LinearComb<java.lang.String>> allCycles)`
  
          Constructs a new flux cap computer from the cycles contained
in the provided fluxome.

`MsFluxIntervalComputer(FbaOptComputer computer,
java.util.Map<java.lang.String,java.util.Set<LinearComb<java.lang.String>>> fluxCaps)`
  
          Constructs a new Mahadevan-Schilling flux interval computer.

`MsFluxIntervalComputer(FbaOptComputer computer,
java.util.Map<java.lang.String,java.util.Set<LinearComb<java.lang.String>>> fluxCaps,
double zeroCutoff)`
  
          Constructs a new Mahadevan-Schilling flux interval computer.

Uses of LinearComb in sbrt.kernel.fba.proc

| Methods in sbrt.kernel.fba.proc that return LinearComb | |
| --- | --- |
| `LinearComb<java.lang.String>` | `FbaOptimizationProcess.getObjectiveFunction()`             Returns the current objective function. |

| Methods in sbrt.kernel.fba.proc with parameters of type LinearComb | |
| --- | --- |
| `void` | `FbaOptimizationProcess.setObjectiveFunction(LinearComb<java.lang.String> objective)`             Sets the objective function to the provided mathematical expression. |

| Method parameters in sbrt.kernel.fba.proc with type arguments of type LinearComb | |
| --- | --- |
| `void` | `RandomObjectiveGenerator.run(ResultsManager<LinearComb<java.lang.String>> resultsManager)`             Generates random objective functions and adds them to the provided results manager. |
| `void` | `FluxCapProcess.run(ResultsManager<java.util.Map<java.lang.String,java.util.Set<LinearComb<java.lang.String>>>> resultsManager)`             Computes the flux caps and adds the result to the provided manager. |

Uses of LinearComb in sbrt.kernel.math

| Classes in sbrt.kernel.math that implement LinearComb | |
| --- | --- |
| `class` | `AbstractLinearComb<V>`             This abstract class is a skeleton implementation of `LinearComb`. |
| `class` | `HashLinearComb<V>`             This class is a concrete implemenation of `LinearComb`. |

| Methods in sbrt.kernel.math that return LinearComb | |
| --- | --- |
| `LinearComb<V>` | `LinearComb.add(double constant)`             Adds, in the mathematical sense, the provided constant to this linear combination. |
| `LinearComb<V>` | `LinearComb.add(LinearComb<? extends V> linComb)`             Adds, in the mathematical sense, the provided linear combination to this linear combination. |
| `LinearComb<V>` | `LinearComb.multiply(double constant)`             Multiplies this linear combination by the provided constant. |
| `LinearComb<V>` | `LinearComb.plugIn(java.util.Map<?,? extends LinearComb<? extends V>> linearCombs)`             Plugs in the provided linear combinations to produce a new linear combination. |
| `LinearComb<V>` | `LinearComb.plugIn(java.util.Map<?,java.lang.Double> numbers, java.util.Map<?,? extends LinearComb<? extends V>> linearCombs)`             Plugs in the provided numerical values and linear combinations to produce a new linear combination. |
| `LinearComb<V>` | `LinearComb.plugIn(java.lang.Object variable, double constant)`             Assigns the value of the provided variable to the provided constant. |
| `LinearComb<V>` | `LinearComb.plugIn(java.lang.Object variable, LinearComb<? extends V> expr)`             Assigns the value of the provided variable to the provided linear combination. |
| `LinearComb<V>` | `LinearComb.plugInNumbers(java.util.Map<?,java.lang.Double> numbers)`             Plugs in the provided numerical values to produce a new linear combination. |
| `LinearComb<V>` | `LinearComb.solveFor(java.lang.Object variable, double constant)`             Equates this linear combination to the provided constant and solves for the provided variable. |

| Methods in sbrt.kernel.math with parameters of type LinearComb | |
| --- | --- |
| `LinearComb<V>` | `LinearComb.add(LinearComb<? extends V> linComb)`             Adds, in the mathematical sense, the provided linear combination to this linear combination. |
| `HashLinearComb<V>` | `HashLinearComb.add(LinearComb<? extends V> expression)`             Adds, in the mathematical sense, the provided linear combination to this linear combination. |
| `LinearComb<V>` | `LinearComb.plugIn(java.lang.Object variable, LinearComb<? extends V> expr)`             Assigns the value of the provided variable to the provided linear combination. |
| `HashLinearComb<V>` | `HashLinearComb.plugIn(java.lang.Object variable, LinearComb<? extends V> expr)`             Plugs the provided linear combination into the provided variable to produce a new linear combination. |

| Method parameters in sbrt.kernel.math with type arguments of type LinearComb | |
| --- | --- |
| `LinearComb<V>` | `LinearComb.plugIn(java.util.Map<?,? extends LinearComb<? extends V>> linearCombs)`             Plugs in the provided linear combinations to produce a new linear combination. |
| `HashLinearComb<V>` | `HashLinearComb.plugIn(java.util.Map<?,? extends LinearComb<? extends V>> variableValues)`             Plugs in the provided linear combinations to produce a new linear combination. |
| `LinearComb<V>` | `LinearComb.plugIn(java.util.Map<?,java.lang.Double> numbers, java.util.Map<?,? extends LinearComb<? extends V>> linearCombs)`             Plugs in the provided numerical values and linear combinations to produce a new linear combination. |
| `HashLinearComb<V>` | `HashLinearComb.plugIn(java.util.Map<?,java.lang.Double> numbers, java.util.Map<?,? extends LinearComb<? extends V>> linearCombs)`             Plugs in the provided numerical values and linear combinations to produce a new linear combination. |

Uses of LinearComb in sbrt.kernel.math.algebra

| Methods in sbrt.kernel.math.algebra that return LinearComb | |
| --- | --- |
| `LinearComb<V>` | `HashLinearSystemSolution.getSolution(java.lang.Object variable)`             Returns the linear combination of free variables to which the provided variable is equal. |
| `LinearComb<V>` | `LinearSystemSolution.getSolution(java.lang.Object variable)`             Returns the linear combination of free variables to which the provided variable is equal. |

| Methods in sbrt.kernel.math.algebra that return types with arguments of type LinearComb | |
| --- | --- |
| `java.util.List<LinearComb<C>>` | `DoubleMatrixWrap.toLinearCombs()`             Returns this matrix as a list of linear combinations. |
| `java.util.List<LinearComb<C>>` | `DoubleMatrix.toLinearCombs()`             Returns this matrix as a list of linear combinations. |
| `java.util.List<LinearComb<C>>` | `AbstractSmdMatrix.toLinearCombs()`             Returns this matrix as a list of linear combinations. |
| `java.util.Map<V,LinearComb<V>>` | `HashLinearSystemSolution.toMap()`             Returns this solution as a map. |
| `java.util.Map<V,LinearComb<V>>` | `LinearSystemSolution.toMap()`             Returns this solution as a map. |

| Method parameters in sbrt.kernel.math.algebra with type arguments of type LinearComb | |
| --- | --- |
| `LinearSystemSolution<V>` | `LinearSystemSolver.solve(java.util.Map<LinearComb<V>,java.lang.Double> equations)`             Computes and returns a solution to the provided system of linear equations. |

Constructor parameters in sbrt.kernel.math.algebra with type arguments of type LinearComb

`HashLinearSystemSolution(java.util.Map<? extends V,? extends LinearComb<? extends V>> solutions)`
  
          Constructs a new hash code-based linear system solution.

Uses of LinearComb in sbrt.kernel.math.algebra.proc

| Method parameters in sbrt.kernel.math.algebra.proc with type arguments of type LinearComb | |
| --- | --- |
| `void` | `LinearSolverProcess.setEquations(java.util.Map<LinearComb<V>,java.lang.Double> equations)`             Sets the system of linear equations to be solver. |

Uses of LinearComb in sbrt.kernel.math.geom

| Methods in sbrt.kernel.math.geom that return LinearComb | |
| --- | --- |
| `LinearComb<V>` | `ParallelHyperplanes.getPlane()`             Returns the hyperplane. |

Constructors in sbrt.kernel.math.geom with parameters of type LinearComb

`ParallelHyperplanes(LinearComb<V> hyperplane,
Interval bounds)`
  
          Constructs a new pair of bounding parallel hyperplanes.

Uses of LinearComb in sbrt.kernel.math.optimization

| Methods in sbrt.kernel.math.optimization with parameters of type LinearComb | |
| --- | --- |
| `void` | `LinearProgramSolver.setConstraint(LinearComb<? extends C> expression, Interval c)`             Sets a constraint on the provided linear combination of column variables. |
| `void` | `LinearProgramSolver.setObjectiveFunction(LinearComb<? extends C> expression)`             Sets the objective function to the provided linear combination of column variables. |
| `void` | `LinearProgramSolver.setObjectiveFunction(LinearComb<? extends C> expression, OptimizationSense sense)`             Sets the objective function and optimization sense to the provided values. |

Uses of LinearComb in sbrt.shell.io.fba

| Methods in sbrt.shell.io.fba that return types with arguments of type LinearComb | |
| --- | --- |
| `static java.util.Set<LinearComb<java.lang.String>>` | `FluxIntervalInputFile.convertVariables(java.util.Set<java.lang.String> rxnNames)`             Converts a set of reaction names to a set of single-term linear combinations. |
| `UnmodifiableMap<java.lang.String,java.util.Set<LinearComb<java.lang.String>>>` | `FluxCapInputFile.getFluxCaps()`             Returns the flux caps contained in this file. |
| `MapFormat<java.lang.String,java.util.Set<LinearComb<java.lang.String>>>` | `FluxCapInputFile.getMapFormat()`             Returns the map format used to parse this file. |
| `java.util.Iterator<java.util.Map<java.lang.String,java.util.Set<LinearComb<java.lang.String>>>>` | `FluxCapInputFile.iterator()`             Returns an iterator over this file. |

| Method parameters in sbrt.shell.io.fba with type arguments of type LinearComb | |
| --- | --- |
| `static FluxConstraints` | `FluxIntervalInputFile.convertConstraints(java.util.Map<LinearComb<java.lang.String>,Interval> intervals)`             Converts the provided intervals into a `FluxConstraints` object. |

Constructor parameters in sbrt.shell.io.fba with type arguments of type LinearComb

`FluxIntervalInputFile(java.lang.String fileName,
FileFormat fileFormat,
java.util.Set<LinearComb<java.lang.String>> requiredVariables,
MapFormat<LinearComb<java.lang.String>,Interval> mapFormat)`
  
          Constructs a new flux-interval input file.

`FluxIntervalInputFile(java.lang.String fileName,
FileFormat fileFormat,
java.util.Set<LinearComb<java.lang.String>> requiredVariables,
MapFormat<LinearComb<java.lang.String>,Interval> mapFormat)`
  
          Constructs a new flux-interval input file.

Uses of LinearComb in sbrt.shell.mng.algebra

| Methods in sbrt.shell.mng.algebra that return types with arguments of type LinearComb | |
| --- | --- |
| `static java.util.Map<LinearComb<java.lang.String>,java.lang.Double>` | `LinearSolverManager.getEquations(ManagerInput<java.lang.String,java.lang.String> input, java.io.PrintWriter out)`             Returns the set of linear equations contained in the file with the provided name. |
| `static java.util.Map<LinearComb<java.lang.String>,java.lang.Double>` | `LinearSolverManager.getEquations(java.lang.String fileName)`             Returns the set of linear equations contained in the file with the provided name. |
| `static java.util.Map<LinearComb<java.lang.String>,java.lang.Double>` | `LinearSolverManager.getEquations(java.lang.String fileName, java.io.PrintWriter out)`             Returns the set of linear equations contained in the file with the provided name. |

Uses of LinearComb in sbrt.shell.mng.fba

| Methods in sbrt.shell.mng.fba that return LinearComb | |
| --- | --- |
| `static LinearComb<java.lang.String>` | `FbaOptManager.getObjectiveFunction(ManagerInput<java.lang.String,java.lang.String> input, java.io.PrintWriter out, Fluxome fluxome)`             Parses and returns the value for `FbaOptManager.OBJECTIVE_FUNCTION` in the provided manager input. |

| Methods in sbrt.shell.mng.fba that return types with arguments of type LinearComb | |
| --- | --- |
| `static java.util.List<LinearComb<java.lang.String>>` | `FluxCapManager.getCycles(ManagerInput<java.lang.String,java.lang.String> input, java.io.PrintWriter out, Fluxome fluxome)`             Opens the file denoted by `FluxCapManager.CYCLE_FILE` in the provided manager input and returns the list of cycles it contains. |
| `static UnmodifiableMap<java.lang.String,java.util.Set<LinearComb<java.lang.String>>>` | `MsFluxIntervalManager.getFluxCaps(ManagerInput<java.lang.String,java.lang.String> input, java.io.PrintWriter out, Fluxome fluxome)`             Returns the flux caps contained in the file with the name specified by `MsFluxIntervalManager.FLUX_CAP_FILE` in the provided input. |
| `static TextInputFile<LinearComb<java.lang.String>>` | `ObjectiveFunctionManager.getInputFile(ManagerInput<java.lang.String,java.lang.String> input, java.io.PrintWriter out, Fluxome fluxome)`             Returns the file denoted by `ObjectiveFunctionManager.OBJ_FUNCT_FILE` in the provided manager input. |
| `static TextInputFile<LinearComb<java.lang.String>>` | `ObjectiveFunctionManager.getInputFile(java.lang.String fileName, FileFormat format, Fluxome fluxome)`             Returns the input file with the provided name. |
| `static TextInputFile<LinearComb<java.lang.String>>` | `ObjectiveFunctionManager.getInputFile(java.lang.String fileName, Fluxome fluxome)`             Returns the input file with the provided name. |
| `static TextOutputFile<LinearComb<java.lang.String>>` | `RandomObjectiveManager.getOutputFile(ManagerInput<java.lang.String,java.lang.String> input, java.io.PrintWriter out, IrrevRxnFluxome fluxome)`             Returns an empty output file that is suitable for storing randomly generated objective functions. |
| `static TextOutputFile<LinearComb<java.lang.String>>` | `RandomObjectiveManager.getOutputFile(java.lang.String fileName, FileFormat format, java.io.PrintWriter out, IrrevRxnFluxome fluxome)`             Returns an empty output file that is suitable for storing randomly generated objective functions. |
| `static RxnNameExprFormat<LinearComb<java.lang.String>,java.lang.String>` | `FbaManager.getRxnNameExprFormat(Fluxome f)`             Returns the default reaction name expression format. |

| Method parameters in sbrt.shell.mng.fba with type arguments of type LinearComb | |
| --- | --- |
| `static FluxIntervalInputFile` | `IntervalVariationManager.getIntervalInputFile(java.lang.String fileName, Fluxome fluxome, MapFormat<LinearComb<java.lang.String>,Interval> mapFormat)`             Returns the flux interval input file with the provided name. |

Uses of LinearComb in sbrt.shell.mng.utilities

| Methods in sbrt.shell.mng.utilities that return types with arguments of type LinearComb | |
| --- | --- |
| `static java.util.List<LinearComb<java.lang.String>>` | `VariableParticipationManager.getExpressions(ManagerInput<java.lang.String,java.lang.String> input, java.io.PrintWriter out)`             Opens the file denoted by `VariableParticipationManager.EXPRESSION_FILE` in the provided manager input and returns the list of mathematical expressions it contains. |
| `VariableParticipation<LinearComb<java.lang.String>,java.lang.String>` | `VariableParticipationManager.getProcess()`             Returns the process this process manager controls. |

Uses of LinearComb in sbrt.shell.text.fba

| Methods in sbrt.shell.text.fba that return LinearComb | |
| --- | --- |
| `LinearComb<java.lang.String>` | `PalssonRxnNodeParser.getProducts(org.w3c.dom.Node reactionNode)`             Returns the products of the provided reaction node. |
| `LinearComb<java.lang.String>` | `BiggRxnNodeParser.getProducts(org.w3c.dom.Node reactionNode)`             Returns the products of the provided reaction node. |
| `LinearComb<java.lang.String>` | `PalssonRxnNodeParser.getReactants(org.w3c.dom.Node reactionNode)`             Returns the reactants of the provided reaction node. |
| `LinearComb<java.lang.String>` | `BiggRxnNodeParser.getReactants(org.w3c.dom.Node reactionNode)`             Returns the reactants of the provided reaction node. |
| `LinearComb<java.lang.String>` | `RxnNameExprFormatV1.parse(java.lang.String rxnNameExpr)`             Parses the provided string and returns its corresponding linear combination of reaction names. |

| Methods in sbrt.shell.text.fba that return types with arguments of type LinearComb | |
| --- | --- |
| `PipeListFormat<java.util.Set<LinearComb<java.lang.String>>>` | `FluxCapFormatV1.getListFormat()`             Returns the format used for lists of values. |
| `PipeListFormat<LinearComb<java.lang.String>>` | `FluxomeSolFileLineFormatV1.getListFormat()`             Returns the format used for lists of values. |
| `PipeSetFormat<LinearComb<java.lang.String>>` | `ConstraintsFileLineFormatV2.getSetFormat()`             Returns the format used for sets of variables. |

| Methods in sbrt.shell.text.fba with parameters of type LinearComb | |
| --- | --- |
| `java.lang.String` | `RxnNameExprFormatV1.format(LinearComb<java.lang.String> rxnNameExpr)`             Returns a formatted string representation of the provided linear combination of reaction names. |

Uses of LinearComb in sbrt.shell.text.math

| Methods in sbrt.shell.text.math that return LinearComb | |
| --- | --- |
| `LinearComb<V>` | `LinearCombFormat.parse(java.lang.String expression)`             Parses the provided string and returns its corresponding linear combination. |
| `LinearComb<V>` | `LinearCombFormatV2.parse(java.lang.String expression)`             Parses the provided string and returns its corresponding linear combination. |
| `LinearComb<V>` | `LinearCombFormatV1.parse(java.lang.String expression)`             Parses the provided string and returns its corresponding linear combination. |

| Methods in sbrt.shell.text.math with parameters of type LinearComb | |
| --- | --- |
| `java.lang.String` | `LinearCombFormat.format(LinearComb<V> expression)`             Returns a formatted string representation of the provided linear combination. |
| `java.lang.String` | `LinearCombFormatV2.format(LinearComb<V> expression)`             Returns a formatted string representation of the provided linear combination. |
| `java.lang.String` | `LinearCombFormatV1.format(LinearComb<V> expression)`             Returns a formatted string representation of the provided linear combination. |

---


|  |  |  |
| --- | --- | --- |
| **Overview** \| **Package** \| **Class** \| **Use** \| **Tree** \| **Deprecated** \| **Index** \| **Help** | |  |
| PREV   NEXT | **FRAMES**    **NO FRAMES**     **All Classes** |  |


---
